# BMI, sex and outcomes in hospitalised patients in western Sweden during the COVID-19 pandemic

**DOI:** 10.1038/s41598-022-09027-w

**Published:** 2022-03-22

**Authors:** Martin Lindgren, Triantafyllia Toska, Christian Alex, Christina E. Lundberg, Ottmar Cronie, Annika Rosengren, Martin Adiels, Helen Sjöland

**Affiliations:** 1 Department of Molecular and Clinical Medicine, Institute of Medicine, Sahlgrenska Academy, University of Gothenburg, Gothenburg, Sweden; 2 Department of Medicine Geriatrics and Emergency Medicine, Sahlgrenska University Hospital, Östra Hospital, Region Västra Götaland, Smörslottsgatan 1, 416 85 Gothenburg, Sweden; 3 School of Public Health and Community Medicine, Institute of Medicine, University of Gothenburg, Gothenburg, Sweden

**Keywords:** Cardiology, Diseases, Endocrinology, Health care, Medical research

## Abstract

High body mass index (BMI) is associated with severe COVID-19 but findings regarding the need of intensive care (IC) and mortality are mixed. Using electronic health records, we identified all patients in western Sweden hospitalised with COVID-19 to evaluate 30-day mortality or assignment to IC. Adjusted logistic regression models were used to estimate odds ratios (OR) and 95% confidence intervals (CI) for outcomes. Of totally 9761 patients, BMI was available in 7325 (75%), included in the study. There was a marked inverse association between BMI and age (underweight and normal weight patients were on average 78 and 75 years, whereas overweight and obese were 68 and 62 years). While older age, male sex and several comorbidities associated with higher mortality after multivariable adjustment, BMI did not. However, BMI ≥ 30 kg/m^2^ (OR 1.46, 95% CI 1.21–1.75) was associated with need of IC; this association was restricted to women (BMI ≥ 30; OR 1.96 (95% CI 1.41–2.73), and not significant in men; OR 1.22 (95% CI 0.97–1.54). In this comprehensive hospital population with COVID-19, BMI was not associated with 30-day mortality risk. Among the obese, women, but not men, had a higher risk of assignment to IC.

## Introduction

The coronavirus SARS-CoV-2 pandemic has imposed considerable strain on health care and emergency services globally. High body mass index (BMI) has been identified as an important risk factor for severe coronavirus SARS-CoV-2 infection defined as need of intensive care (IC) or death^1–4^. However, the role of an elevated BMI in severity and mortality in hospitalised patients has still not been entirely clarified, with several systematic reviews and large cohort studies showing conflicting results^5–11^. To some degree, this may reflect variations between countries with respect to interventions limiting community spread of virus, access to health care, and the age-distribution of the population.

Advanced age, male sex, diabetes type 2, and pre-existing cardiovascular disease (CVD) have been firmly established as risk factors for adverse outcomes^12–16^. Additionally, overweight and obesity are known to be associated with more severe disease, in particular need of IC, while findings with respect to mortality have been more heterogeneous^5–11^. A recent Swedish study based on over 1600 patients treated in IC units, out of which 39% were obese, found elevated BMI to be associated with higher mortality risk^17^. However, patients admitted to dedicated IC units in Sweden are generally undergoing careful evaluation of their prospective likelihood of a favourable outcome and benefit of therapies prior to admission, and are on average younger than other hospitalised patients. Patients treated in IC units for COVID-19 are also more often men, with only one in four Swedish admitted patients being female, while the proportion of women among all hospitalised patients is higher^18^.

The obesity paradox implies that negative effects of many disorders are reduced with higher BMI, with more severe outcomes in patients with normal or low weight^19^. Because many patients hospitalised with severe COVID-19 are elderly, being underweight or normal weight might be associated with poorer outcomes in patients who are not considered for IC because of advanced age and frailty, but this has been sparsely studied in COVID-19 patients.

Few studies have simultaneously considered the impact of BMI on the use of IC procedures and mortality in unselected patients hospitalised with COVID-19. To this end we included all cases of COVID-19 requiring in-hospital treatment, from an entire region of Sweden, describing their pre-covid health and analysing outcome and level of in-hospital care (assignment to IC) relative to BMI. We report the outcome measures of death within 30 days of admission or need of IC, where the latter was defined as mechanical ventilation by endotracheal tube or high flow nasal oxygen treatment (HFOT) in relation to age, sex and BMI. Data on all hospitalised patients was available from the collected electronic health records (EHRs) of the Region Västra Götaland (VGR) in Sweden, covering all hospital and a sizable proportion of primary care in 1.7 million inhabitants during 02/01/2020 to 06/21/2021. During the course of the pandemic, the availability of HFOT and the evidence of its favourable effects grew rapidly. In Sweden this was followed by speedy temporary conversion of hospital beds in regular wards to providers of HFOT, organized outside traditional IC units. Patients treated in such units are also included in our study in addition to patients treated in traditional IC units.

## Methods

### Study design, population and observation period

Information on the study population was derived from a comprehensive regional database of EHRs, covering all patients with a confirmed diagnostic test and first diagnosis of COVID-19. Patients were admitted as in-patients to a regional emergency medical service, with a registered COVID-19-related diagnosis from ICD-10; U071 or U 072. We included all patients 18 years and older admitted for in-hospital care who were registered in the VGR in western Sweden and alive on February 1, 2020. The inclusion period ranged from February 1, 2020, to June 21, 2021. Outcomes included (1) assignment to IC at any time during hospitalisation and (2) death within 30 days.

### Data source and participants

We used data from a regional comprehensive database of EHRs covering all hospital admissions, out-patient specialist care and primary care visits in public healthcare in the VGR region of Sweden, since January 1, 2017. Private outpatient specialist and primary care visits are not covered. Based on the number of private care operators in the VGR the outpatient data are estimated to include 70% of all citizens (approximately 1.2 million). Deaths were captured through linkage to the national Cause-Specific Death Register. Patient data were pseudonymized upon extraction from the region VGR database for analysis, minimizing the risk for violation of integrity and reidentification. The structure of the database allowed for near real time extraction of clinical data and outcomes.

### Regional community and health care system

The VGR houses just over 1.7 million (approximately 17% of the Swedish population) citizens, and contains eight hospitals with in-patient emergency services, seven county hospitals, and one university hospital (Sahlgrenska University Hospital), together holding approximately 3000 hospital beds, and 200 publicly financed primary care centres, representing all emergency hospital services and 57% of primary care services. During 2019, 80% of the population was registered as having at least one contact with a care provider.

### Outcomes and variables assessed

The outcomes of this study were (1) death within 30 days after admission, or (2) admittance to IC defined as mechanical ventilation by endotracheal intubation (identified by the ICD-10 procedure code DG017 or DG018) or use of HFOT (DG028, Heated humidified high-flow nasal therapy). Outcome assessment was based on retrieval of data recorded in routine health care, registered in the database. Previously registered cardiometabolic diseases were retrieved by ICD-codes; diabetes type 1 (E10), diabetes type 2 (E11), dyslipidaemia (E78), obesity (E66), hypertension (I10.9), atrial fibrillation (I48.9), heart failure (I50), chronic obstructive pulmonary disease (COPD) (J44), asthma (J45), chronic kidney disease (N18) and ischemic stroke (I63). For the following vital signs, a first recorded value on the date of hospital admission on admission was retrieved; heart rate (beats per minute), temperature (degrees Celsius), respiratory rate (breaths/minute), systolic and diastolic blood pressure (mmHg) and oxygen saturation (%).

### Statistical analysis

The follow-up period started at the date of hospital admission (baseline), and subjects were followed until need of IC as defined above, or death within 30 days. BMI, calculated as weight in kg divided by height in m squared (kg/m^2^), was divided into four categories (BMI < 18.5, 18.5 to < 25, 25 to < 30, and ≥ 30), with BMI 18.5 to < 25 as reference. Age was divided into five categories; 18 to 54, 55 to 64, 65 to 74, 75 to 84, and ≥ 85 years. Logistic regression models were used to estimate odds ratios (OR) and 95% confidence intervals (CI) regarding the association between outcomes and clinical variables, with emphasis on BMI, age and sex, while adjusting for potential confounders. Model 1 was adjusted for age and sex. Model 2 was further adjusted for comorbidities registered at baseline. We present both models in total, and stratified for sex. In addition, a sensitivity analysis with Model 2 was performed for all individuals with RC (where all individuals with IC were excluded) with death as an outcome. Furthermore, spline plots were generated based on an age adjusted logistic regression model, stratified by sex, with BMI as a restricted cubic spline with knots placed at 5^th^, 35^th^, 65^th^, and 95^th^ percentiles. BMI values between 15 and 50 were plotted against the reference (BMI 20). Statistical calculations were performed using R ver. 4.0.3 software (http://www.R-project.org).

### Ethics statement

The study conforms to the principles outlined in the Declaration of Helsinki. The study was approved and informed consent from the study participants was waived by the Swedish Ethical Review Authority, through the Ethics Committee of the University of Lund (EPN Reference: DNR 2020-02020).

## Results

### Study population

**Figure 1:**
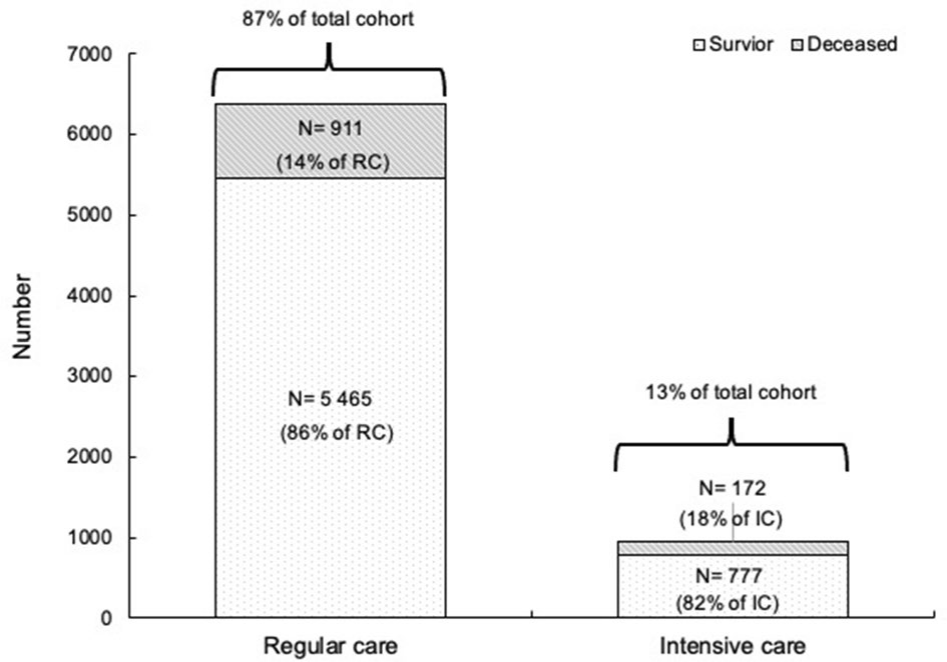
Number and percent of population who survived and died by regular care vs. intensive care. A considerable majority of all hospitalized patients were treated in regular care and survived. *RC* regular care, *IC* intensive care.

We identified 9761 patients with a diagnosis of COVID-19 admitted to a regional hospital fulfilling the definitions of the cohort with BMI measurements available in 7325 (75%), who form the analytical cohort of the present study (Supplementary Fig. 1). Baseline characteristics are presented in Table 1, in total and stratified into BMI categories. The median age of all patients was 68 years (inter-quartile range [IQR] 55, 79), 41% were women, and 13% received IC (Fig. 1). Median age in the two lowest BMI categories (BMI kg/m^2^ < 25) was generally higher (median 78 [IQR 67, 87] and 75 [IQR 61, 84] years, respectively), whereas for BMI kg/m^2^ ≥ 30, the median age was lower (median 62 years [IQR 50, 74]), and the numerically largest group (BMI kg/m^2^ 25 to < 30) had a median age of 68 years [IQR 55, 79]. In the BMI kg/m^2^ < 18.5 category, one in three (34%) was 85 years and older, whereas for BMI kg/m^2^ ≥ 30, the youngest age group dominated (33%). The overall most common comorbidities were hypertension (44%) and dyslipidemia (24%). Several comorbidities were more prevalent with higher BMI: dyslipidemia (ranged from 20% to 26%, from lowest to highest BMI category), diabetes mellitus type 2 (ranged from 11% to 28%), and asthma (ranged from 4.5% to 12%), whereas COPD was less prevalent (from 19% to 6.4%). Vital signs on admission were generally evenly distributed across BMI groups, with normal body temperature, blood pressure and heart rate, but elevated respiratory rate and lower oxygen saturation. Data on vital signs during day of admission were missing in 30–40% of the patients. Comorbidity data were retrieved from previously recorded diagnostic codes in EHRs, and the prevalence of obesity did thus not reflect the classification from BMI-measurements.

**Table 1:** Demographic data of study participants by body mass index categories.

Group	Total	BMI < 18.5	BMI 18.5 to < 25	BMI 25 to < 30	BMI ≥ 30
N	7325	222	2193	2615	2295
Age (years), median (IQR)	68 (55, 79)	78 (67, 87)	75 (61, 84)	68 (55, 79)	62 (50, 74)
**Age group, n (%)**					
18 to 54	1818 (25)	31 (14)	391 (18)	634 (24)	762 (33)
55 to 64	1329 (18)	17 (7.7)	259 (12)	530 (20)	523 (23)
65 to 74	1435 (20)	44 (20)	406 (19)	507 (19)	478 (21)
75 to 84	1624 (22)	54 (24)	600 (27)	590 (23)	380 (17)
85 to 110	1119 (15)	76 (34)	537 (24)	354 (14)	152 (6.6)
**Sex, n (%)**					
Women	2986 (41)	130 (59)	919 (42)	916 (35)	1021 (44)
**Comorbidities, n (%)**					
Hypertension	3218 (44)	99 (45)	959 (44)	1100 (42)	1060 (46)
Dyslipidemia	1755 (24)	44 (20)	482 (22)	627 (24)	602 (26)
Diabetes type I	215 (2.9)	7 (3.2)	62 (2.8)	71 (2.7)	75 (3.3)
Diabetes type II	1564 (21)	24 (11)	389 (18)	519 (20)	632 (28)
Obesity	998 (14)	2 (0.9)	42 (1.9)	198 (7.6)	756 (33)
Cardiovascular disease	966 (13)	31 (14)	322 (15)	344 (13)	269 (12)
Atrial fibrillation	1017 (14)	34 (15)	361 (16)	355 (14)	267 (12)
Heart Failure	773 (11)	27 (12)	261 (12)	245 (9.4)	240 (10)
COPD	556 (7.6)	42 (19)	216 (9.8)	151 (5.8)	147 (6.4)
Asthma	701 (9.6)	10 (4.5)	188 (8.6)	233 (8.9)	270 (12)
Chronic kidney disease	522 (7.1)	19 (8.6)	184 (8.4)	188 (7.2)	131 (5.7)
Ischemic stroke	307 (4.2)	8 (3.6)	120 (5.5)	107 (4.1)	72 (3.1)
Dementia	256 (3.5)	19 (8.6)	118 (5.4)	76 (2.9)	43 (1.9)
**Initial vital signs, median (IQR)**					
Heart rate	84 (75, 90)	80 (72, 90)	81 (72, 90)	84 (74, 90)	86 (78, 92)
Temperature	37.50 (36.80, 38.30)	37.10 (36.60, 37.95)	37.40 (36.70, 38.20)	37.50 (36.80, 38.30)	37.70 (37.00, 38.50)
Respiratory rate	22 (18, 26)	20 (16, 24)	20 (18, 26)	22 (19, 28)	22 (20, 28)
Systolic blood pressure	130 (118, 145)	130 (114, 143)	127 (114, 145)	130 (119, 145)	131 (120, 145)
Diastolic blood pressure	77 (69, 85)	75 (64, 86)	75 (66, 82)	78 (70, 85)	79 (70, 86)
Saturation (%)	95.0 (92.0, 97.0)	95.0 (92.0, 98.0)	95.0 (92.0, 98.0)	95.0 (92.0, 97.0)	94.0 (91.0, 97.0)

*BMI* body mass index, *COPD* chronic obstructive pulmonary disease, *IQR* inter quartile range.

**Figure 2:**
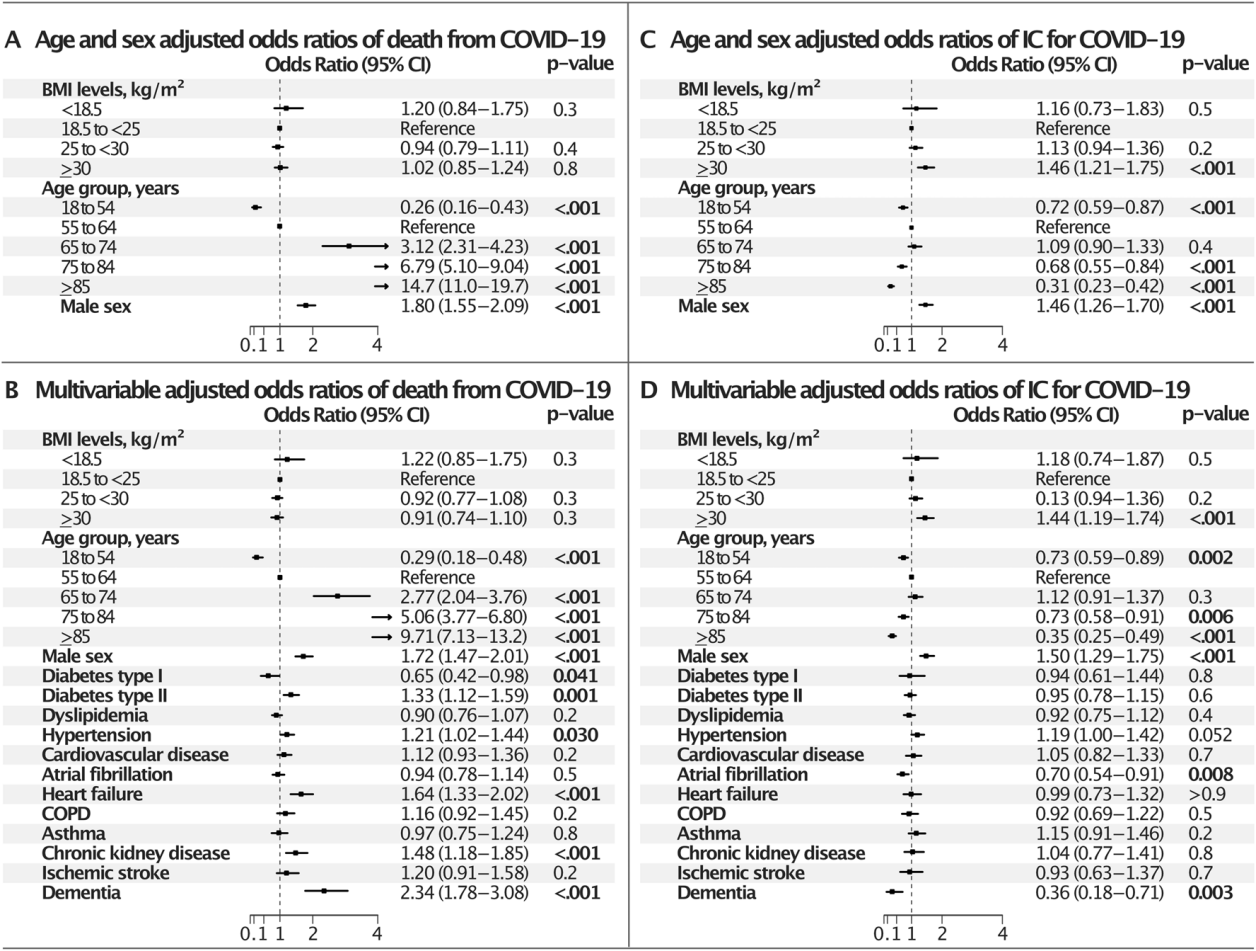
Age- and sex adjusted and multivariable adjusted odds ratios of IC and death from Covid 19. *COPD* chronic obstructive pulmonary disease, *IC* intensive care. **(A)** Age- and sex adjusted odds ratios of death, **(B)** multivariable adjusted odds ratios of death, **(C)** age- and sex adjusted odds ratios of IC, **(D)** multivariable adjusted odds ratios of IC.

Overall 30-day mortality was 14.8%. Figure 1 shows the distribution between RC/IC in the study population and their respective mortality (RC/IC 14.3%/18.1%), resulting in 15.9% of all deaths occurring in IC. Figure 2 shows the adjusted ORs for death and IC from COVID-19 (< 30 days). Adjusted for age and sex, (Fig. 2A) BMI did not associate with death. Age associated with death (p < 0.001 for all age groups, compared to the reference group (55–64 years)). The youngest age group (18–54 years) displayed the lowest odds of death. The odds for death increased with each successive age group and with male sex. After multivariable adjustment (Fig. 2B) the odds pattern for mortality persisted for BMI, age and male sex, with the odds in the oldest patients partially attenuated (OR 9.71, CI 7.13–13.2, age ≥ 85 years). Among comorbidities, diabetes type 2, hypertension, heart failure, chronic kidney disease, and dementia were associated with death, the association being most pronounced for dementia (OR 2.34, CI 1.78–3.08), whereas diabetes type 1 was associated with lower odds (OR 0.65, CI 0.42–0.98). For level of care, adjusted for age and sex, BMI ≥ 30 associated with increased risk of IC (Fig. 2C) (OR 1.46, CI 1.21–1.75) as did male sex (OR 1.46, CI 1.26–1.70). The youngest and oldest age groups were associated with lower odds of IC (p < 0.001 for all). After multivariable adjustment (Fig. 2D) the association between BMI ≥ 30 and IC remained (OR 1.44, CI 1.19–1.74) and all other ORs were sustained at similar levels. Also, atrial fibrillation and dementia associated with reduced odds of IC (OR 0.70, CI 0.54–0.91, and OR 0.36, CI 0.18–0.71, respectively).

Men and women differed at baseline (Supplementary Tables 1 and 2). Inclusion of interactions between BMI and sex in the models showed significant interactions for IC as outcome (Supplementary Table 3) but not for mortality (Supplementary Table 4). Therefore sex specific models were developed.

**Figure 3:**
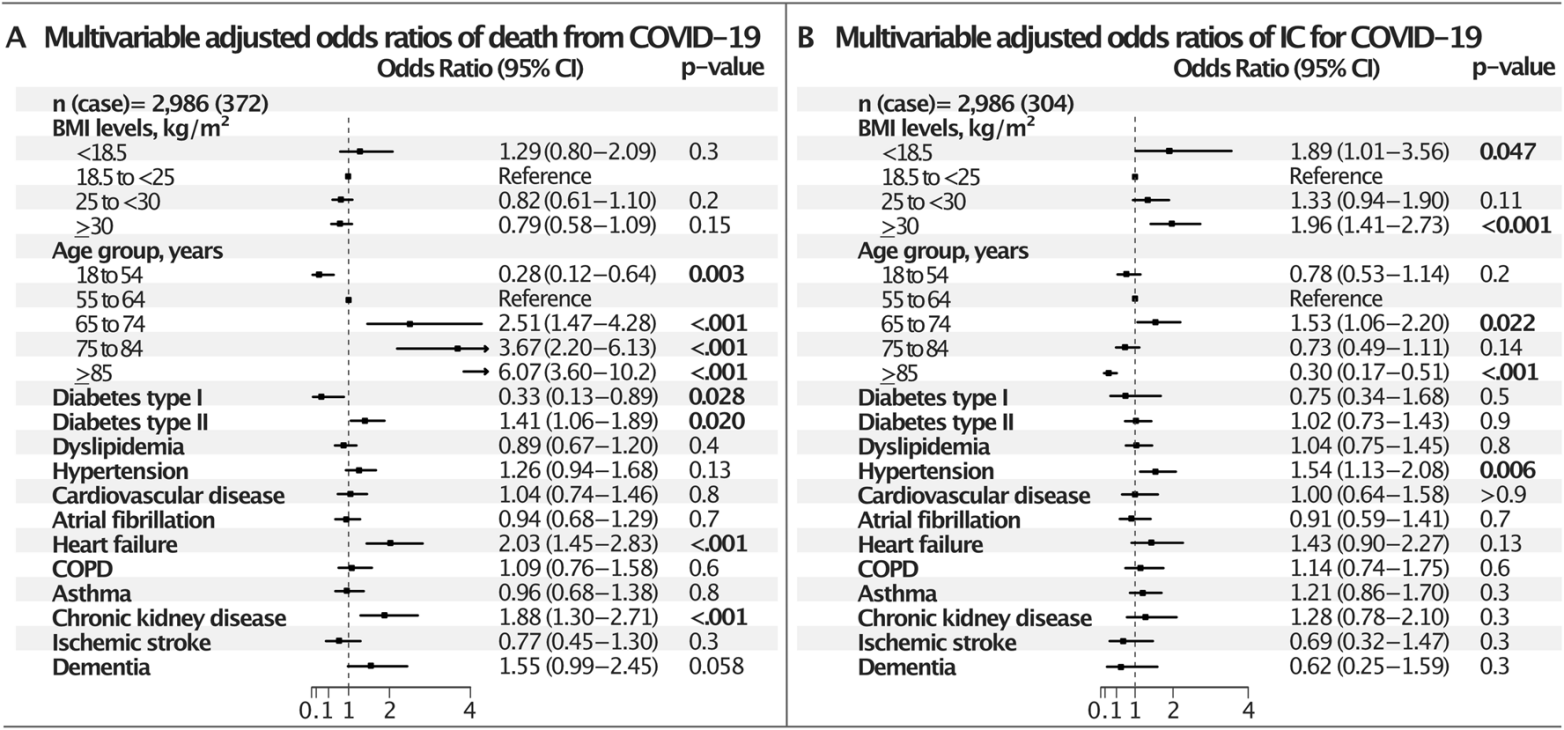
Multivariable adjusted odds ratios of IC and death from COVID-19, in women. *COPD* chronic obstructive pulmonary disease, *IC* intensive care. **(A)** Multivariable adjusted odds ratios of death, **(B)** multivariable adjusted odds ratios of IC.

**Figure 4:**
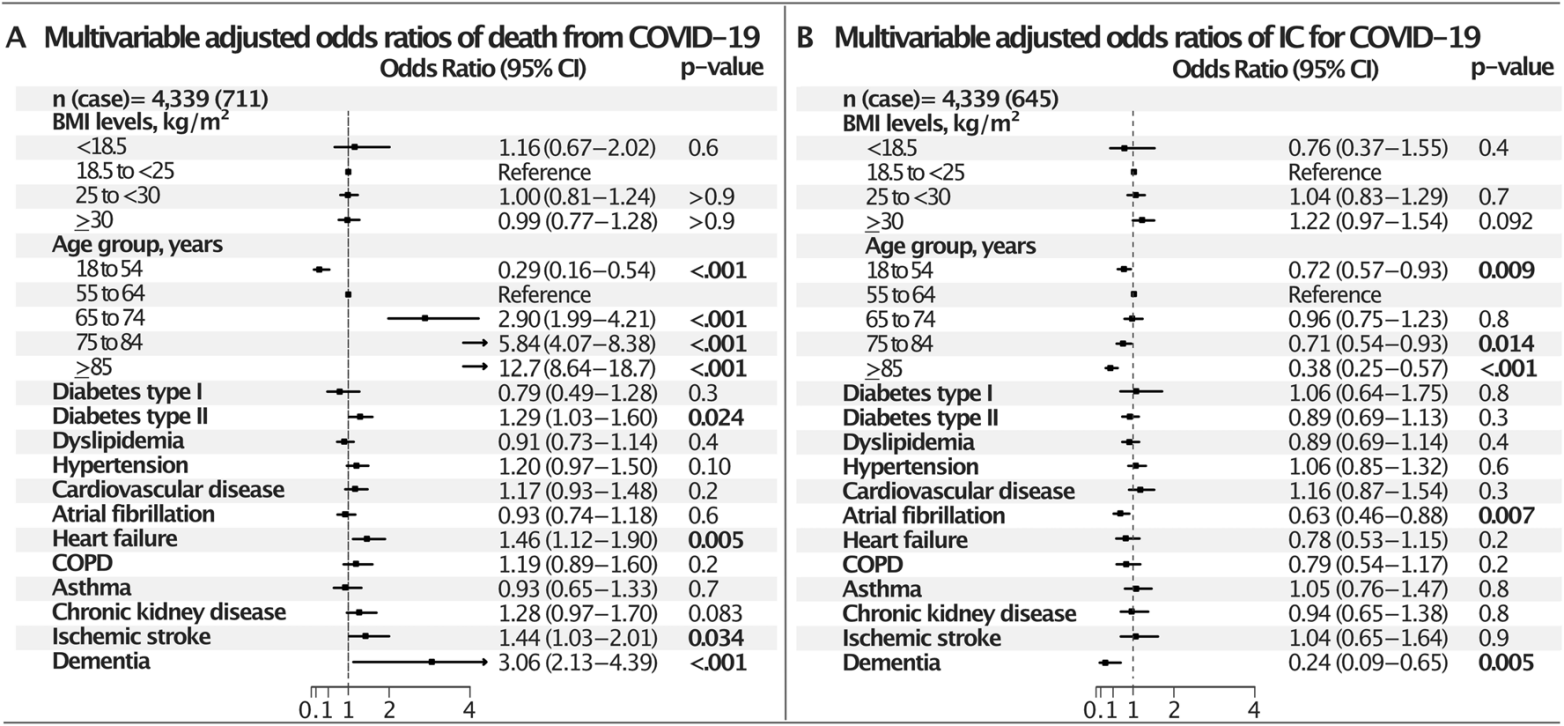
Multivariable adjusted odds ratios of IC and death from COVID-19, in men. *COPD* chronic obstructive pulmonary disease, *IC* intensive care. **(A)** Multivariable adjusted odds ratios of death, **(B)** multivariable adjusted odds ratios of IC.

The adjusted outcome measures for women and men are presented in Figs. 3 and 4. In women (Fig. 3) the association between BMI and IC was U-shaped; the odds for BMI ≥ 30 was OR 1.96 (CI 1.41–2.73) compared to the reference group, while the OR for BMI < 18.5 was 1.89 (CI 1.01–3.56) after multivariable adjustment. The only comorbidity independently predictive of IC in women was hypertension, OR 1.54 (CI 1.13–2.08). Being very old was a negative predictor. There was no association between BMI and mortality, whereas heart failure, chronic kidney disease and diabetes type 2 were independently predictive of death. Type 1 diabetes appeared to be protective: OR 0.33 (CI 0.13–0.89).

In men (Fig. 4) BMI did not associate with death or IC. As for women, heart failure and diabetes type 2 were independently predictive of death. Patients admitted to hospital without available measurements for BMI are presented in Supplementary Table 5 (n = 2432) and were generally younger (41% in age cohort 18–54), with fewer comorbidities, less affected vital parameters and lower mortality and assignment to IC. A sensitivity analysis of adjusted odds ratios of death for patients who were in RC only, were similar to outcome for the whole population, except that the protective effect in the youngest women was no longer significant, whereas a similar odds ratio for chronic kidney disease in men reached significance (p = 0.047) (Supplementary Table 6). Men and women differed at baseline (Supplementary Tables 1 and 2) as women were older in all BMI cohorts, men suffered more often diabetes in the lowest BMI cohort and cardiovascular disease in the obese cohort. COPD among women was disproportionally located to BMI < 18.5 and asthma to BMI ≥ 25 groups. Logistic regression models for estimation of odds risk were adjusted for age, sex and comorbidities.

**Figure 5:**
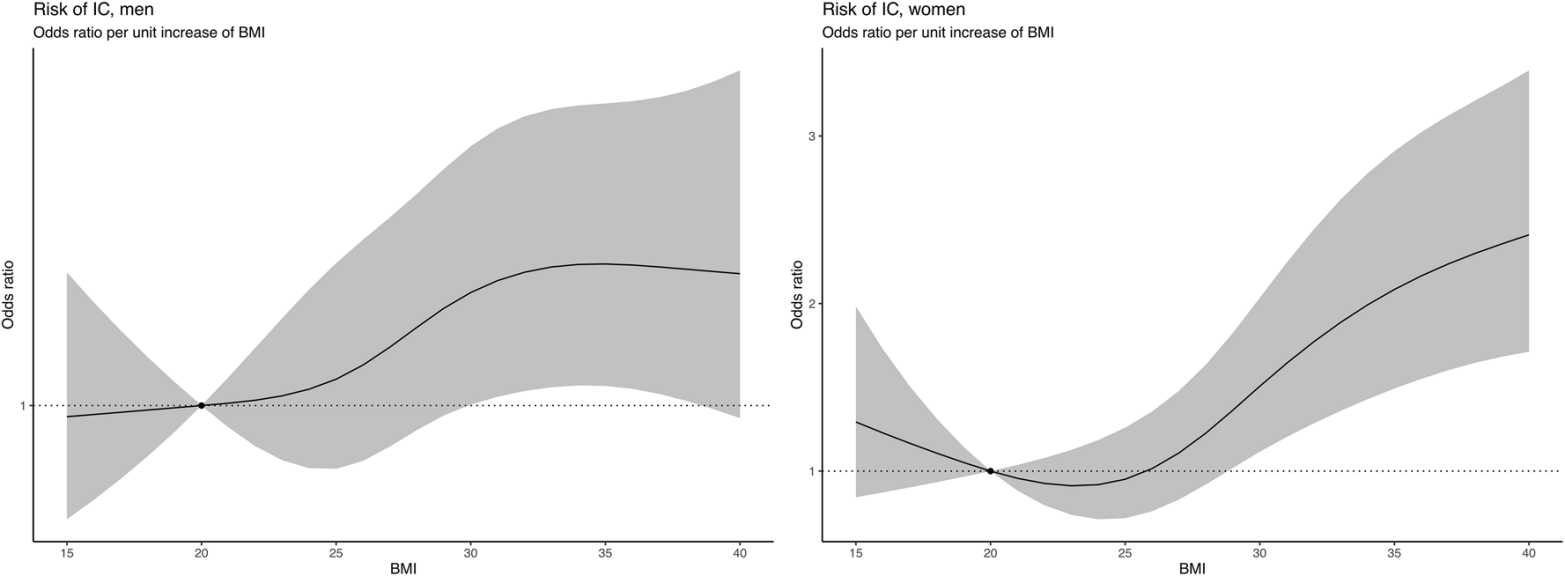
Age adjusted odds ratios (95% CI) of IC by unit of increase in BMI with BMI 20 kg/m^2^ as a reference, for men (left) and women (right). *BMI* body mass index, *IC* intensive care.

For women the association between increasing BMI and risk for treatment in IC was confirmed by spline interpolation, whereas findings in men were inconclusive, and displaying increasing confidence intervals with higher BMI (Fig. 5). For BMI and death no such association was confirmed (Supplementary Fig. 2), although there was a trend towards a J-shaped association between BMI and death for both women and men.

## Discussion

Our database allowed for capture of all cases from the population of region VGR admitted for emergency medical care with a laboratory confirmed diagnosis of COVID-19. In total, we were able to extract information on BMI in 75% of the patients, which were included in the present analysis. In general, baseline findings (Table 1) in hospitalised patients corresponded well with previous observations under similar conditions^6,12^. Thus, the majority of patients were old (> 65 years), more often male, overweight, and with a high prevalence of cardiometabolic comorbidities.

Obese patients were much younger than those who were normal or underweight. In the whole cohort, more than half (56%) of obese patients (BMI ≥ 30) were less than 65 years old, whereas most (57%) of underweight patients (BMI < 18.5) were 65 years or older, probably reflecting general frailty among older persons with severe COVID-19. Other comorbidities and risk factors appeared balanced between groups, except for dyslipidemia and diabetes which were more common in the obese group. Vital signs during the first 24 h were mostly within normal limits, except for elevated respiratory rate and impaired oxygen saturation, which were most prevalent in the obese subcohort. Among vital signs, blood pressure and heart rate did not vary between the different BMI classes, which may be carefully interpreted as an indication of comparable treatment regimens across BMI classes for cardiovascular risk factors and similarities in overall cardiovascular health status. However, the phenotype of diabetes, differences in detailed treatment regimens or duration of disease cannot be understood from blood pressure and heart rate, and interpretations of similarities must be made with caution. Although characteristics of included patients corresponded with other observations of hospitalised populations, the distribution with respect to age, BMI and underlying disease also reflect the composition of the background population. This will also be influenced by factors such as community health, health care system, community spread of infection, and distribution of age in the population. The exact composition of hospitalised patients will thus vary between countries.

In the complete cohort, only a minority of patients were assigned to IC, with higher mortality (18%) in IC compared with RC (14%). However, the majority of deaths (84.1%) took place in RC, which is likely to reflect a population that was unable to tolerate HFOT or other IC measures. The skewed distribution of BMI relative to age was likely to covary with BMI and age-related outcomes. Overall, outcomes corresponded well with expected results of increased mortality risk associated with more advanced age, male sex and cardiometabolic comorbidities, confirming a striking excess risk associated with age^13,15,16,20^. The explanations are partly increased frailty and comorbidities, although the very sharp increase in risk with higher age is not completely accounted for. Similarly, the increased risk for death among men has been previously observed but not conclusively explained^13,15,16,20^.

Of note, BMI was not associated with death, a matter previously addressed with contradictory results^4,8–11,21,22^. The relative risk for assignment to IC was inversely associated with age, with a successively smaller proportion of patients assigned to IC with increasing age. This is consistent with standard considerations as comorbidities and frailty increase with age and limit the potential benefits of IC and the individual´s ability to withstand the demands of HFOT or IC.

Our data showed significantly increased odds of IC among patients with BMI ≥ 30. Also, there seemed to be an interaction with sex for women. IC in COVID-19 was generally indicated due to respiratory failure, presented as strenuous respiratory work (elevated breathing frequency) or hypoxia on admission. Circulatory failure was infrequent in our study, and COVID-19 is primarily a respiratory disease. The explanation for the elevated proportion of patients with obesity admitted to IC is likely to be multifactorial. However, overweight and obesity limit respiratory capacity by several mechanisms that can be emphasized: First, altered mechanical properties of the chest wall and lungs due to fat deposits in mediastinum and abdominal cavities will change the normal breathing pattern, and increase the work of breathing^23^. The compliance of lungs and chest wall are affected, and resting lung volumes are reduced by overweight and obesity^23,24^. Also, effects on airway tone and laxity will lead to increased airway closure, increased airway reactivity and lack of uniform ventilation. Obesity is also associated with increased hormonal activity and inflammation, through circulating adipokines and cytokines, directly linked to airway inflammation, hyperresponsiveness and obstruction, and cell damage further compromising respiratory function. The adipogenic hormone leptin, which is increased in obesity, may affect ventilatory drive and airway obstruction. All of the above may contribute to increased symptoms, respiratory failure and more severe course of COVID-19 in the obese^23–26^.

The relative distribution of a higher proportion of obesity in younger patients hospitalised with COVID-19 have been observed previously^27^. Prior studies have confirmed that weight gain and acquisition of obesity earlier in life carries a generally more serious prognosis than development of obesity in later life^28,29^ which is supposedly associated with accelerated development of cardiovascular and metabolic complications^30^. This is supported by hypertension, hyperlipidemia and diabetes being more frequent in the obese subcohort^4,21,23,25^.

The excess risk of obesity in relation to need of IC was observed solely in women; however, in total a third of all patients were obese, which is a higher proportion than in the background population. Prior studies of BMI, obesity and lung function have found a negative association between BMI and lung function, more evident in women than in men^31^. Also, symptomatic breathlessness and reduced lung function among patients with obesity is more prevalent in women^32^. In contrast, men are at a significantly higher risk of developing severe COVID-19, and thus constitute a numerically dominating part of the patients in IC units^17^. However, for influenza, although men are more susceptible to infection, women have more pronounced immune responses once infected, and animal experiments also support a greater inflammatory response, impaired lung tissue repair, and hormonal triggers of pulmonary inflammation in the female sex^33^. We do not know if this is true also for other viral pulmonary infections, such as SARS-CoV-2, in women, but our findings of higher odds of IC treatment for women is consistent with such a mechanism. Finally, a body fat distribution pattern of concentration of central fat (android obesity), is related to more detrimental changes in lung function compared to gynoid obesity (lower body distribution)^27^. However, the obesity pattern for patients in our study is not known, and the contribution of such a mechanism remains speculative. Still, the combination of obesity and female sex appears to carry specific respiratory vulnerability necessitating IC in COVID-19 infection.

### Strengths and limitations

One strength of this study is the comprehensive coverage of hospitalised patients with COVID-19, encompassing complete availability of all patients hospitalised due to a laboratory confirmed diagnosis of COVID-19 from a complete region of western Sweden. Also, the accessibility to extensive clinical data retrieved from EHRs with near real time availability ensured a detailed dataset. Furthermore, we believe that access to all patients treated with IC measures, also outside traditional IC units, in the temporary intermediate units during the pandemic, provides a more comprehensive representation of the hospitalised population with COVID-19. Finally, as a result of linkage between test data and hospital records our dataset included only patients with a laboratory confirmed diagnosis of COVID-19. However, our study has also some weaknesses. For example, BMI and risk factor diagnoses in the background population are not known, and we lack prior out-patient data from primary care delivered by the private sector (approximately 40% of primary care). Measurement of individual contacts with public care for all citizens (during 2 years of hospital and specialized care) gives an estimate of 70% coverage of the population prior to the present hospitalisation. We were able to retrieve BMI data from 75% of patients in total. It is likely that the mere presence of a registration of BMI is associated with a more vulnerable population with health care problems related to either abnormally high or low baseline BMI, which may represent a bias in the study. Also, of considerable interest for the outcome of COVID-19, we had no available measurement of frailty in the cohort, and no data on immigrant status or ethnicity. Furthermore, obstructive sleep apnea (OSA) is an important risk factor associated with BMI, cardiovascular disease and hypoventilation which has been linked to severe COVID-19. However, it did not turn up among the most frequent comorbidities retrieved from EHRs and was thus not included in our study.

Also, the limitations of BMI must be addressed: Its shortcomings include inability to determine body composition, fat mass and allocation of body fat, thereby at risk of overrating obesity in individuals with high muscle mass. Despite its flaws, BMI is still considered the best predictor of unhealthy weight and the most commonly used anthropometrical measure as a proxy for overweight and obesity, recommended by WHO^34^. Its advantages include easy accessibility and well documented association with cardiovascular disease^34,35^. The outcome of hospital admissions and treatment will reflect the composition of the background population (median age, comorbidities, state of nutrition, and prevalence of obesity) and the organization of the health care system. In addition, in the case of a communicable disease, community spread of infection and the speed of application of vaccination programs will affect outcome. Therefore, our results have limited use for comparisons between different health care systems and general organization. Finally, specific medical treatment for COVID-19 is not included in the present analyses, as the focus is directed towards conditions prior to, or early in the course of hospital admission, so as to have association with decision on level of care.

## Conclusions

We studied predictors for mortality and IC in a complete cohort of patients hospitalised with COVID-19 in region VGR, Sweden, with respect to BMI, from a clinical data repository providing high granularity regarding clinical observations and pre-covid conditions. We found that (1) The risk of admittance to IC associated with higher BMI ≥ 30, (2) this risk was only detected in women, (3) BMI did not associate with mortality in hospitalised patients. Our findings provide important information regarding the distribution and need of care in obesity and COVID-19.

## Supplementary Information

Supplementary Information.

## Data Availability

The datasets analysed in the current study are not publicly available due to ethical reasons but are available from the corresponding author on reasonable request.
