# Supplementary material for: BMI, sex and outcomes in hospitalised patients in western Sweden during the COVID-19 pandemic
Source: Sci Rep. 2022 Mar 22;12:4918. doi: 10.1038/s41598-022-09027-w (PMC8939489; doi:10.1038/s41598-022-09027-w)
Supplement: Supplementary file 1 — Supplementary Information. [file 41598_2022_9027_MOESM1_ESM.docx]

**Supplementary Figure 1.** Flowchart for inclusion and exclusion criteria in the study.


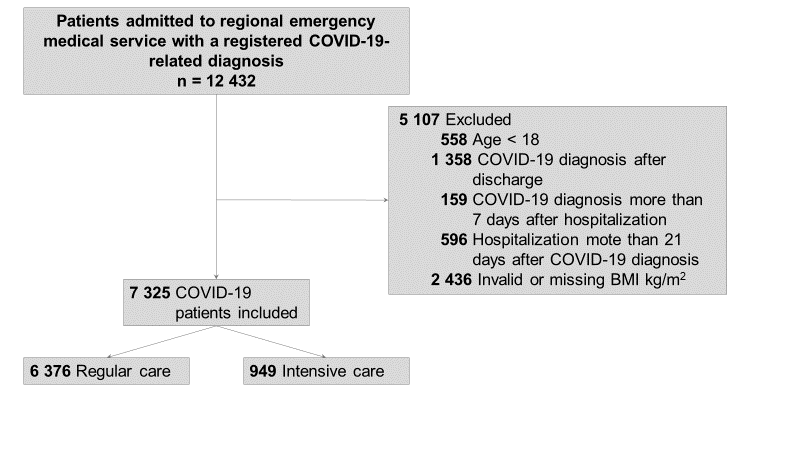


**Supplementary Table 1.** Characteristics of study participants by BMI categories, men.

| Group | Men | BMI <18.5 | BMI 18.5 to <25 | BMI 25 to <30 | BMI >30 |
| --- | --- | --- | --- | --- | --- |
| N | 4,339 | 92 | 1,274 | 1,699 | 1,274 |
| Age, years (IQR) | 67 (55, 78) | 74 (64, 85) | 75 (62, 83) | 66 (55, 77) | 61 (49, 72) |
| **Age group, n (%)** |  |  |  |  |  |
| 18 to 54 | 1,077 (25) | 17 (18) | 193 (15) | 418 (25) | 449 (35) |
| 55 to 64 | 842 (19) | 7 (7.6) | 165 (13) | 379 (22) | 291 (23) |
| 65 to 74 | 915 (21) | 24 (26) | 263 (21) | 353 (21) | 275 (22) |
| 75 to 84 | 974 (22) | 18 (20) | 377 (30) | 381 (22) | 198 (16) |
| >85 | 531 (12) | 26 (28) | 276 (22) | 168 (9.9) | 61 (4.8) |
| **Comorbidities, n (%)** |  |  |  |  |  |
| Hypertension | 1,863 (43) | 39 (42) | 536 (42) | 693 (41) | 595 (47) |
| Dyslipidemia | 1,110 (26) | 20 (22) | 296 (23) | 421 (25) | 373 (29) |
| Diabetes type I | 145 (3.3) | 6 (6.5) | 42 (3.3) | 50 (2.9) | 47 (3.7) |
| Diabetes type II | 991 (23) | 15 (16) | 253 (20) | 359 (21) | 364 (29) |
| Obesity | 541 (12) | 1 (1.1) | 25 (2.0) | 113 (6.7) | 402 (32) |
| Cardiovascular disease | 672 (15) | 14 (15) | 220 (17) | 247 (15) | 191 (15) |
| Atrial fibrillation | 639 (15) | 12 (13) | 220 (17) | 247 (15) | 160 (13) |
| Heart Failure | 472 (11) | 10 (11) | 158 (12) | 167 (9.8) | 137 (11) |
| COPD | 309 (7.1) | 12 (13) | 118 (9.3) | 94 (5.5) | 85 (6.7) |
| Asthma | 320 (7.4) | 2 (2.2) | 88 (6.9) | 110 (6.5) | 120 (9.4) |
| Chronic kidney disease | 335 (7.7) | 8 (8.7) | 111 (8.7) | 141 (8.3) | 75 (5.9) |
| Ischemic stroke | 199 (4.6) | 3 (3.3) | 79 (6.2) | 79 (4.7) | 38 (3.0) |
| Dementia | 151 (3.5) | 7 (7.6) | 74 (5.8) | 48 (2.8) | 22 (1.7) |
| **Initial vital signs, median (IQR)** |  |  |  |  |  |
| Heart rate | 84 (75, 91) | 80 (71, 88) | 82 (72, 90) | 84 (75, 91) | 87 (79, 93) |
| Temperature | 37.60 (36.80, 38.40) | 37.20 (36.60, 38.10) | 37.40 (36.70, 38.20) | 37.60 (36.80, 38.30) | 37.80 (37.00, 38.60) |
| Respiratory rate | 22 (19, 28) | 20 (18, 24) | 21 (18, 26) | 22 (20, 28) | 23 (20, 28) |
| Systolic blood pressure | 130 (119, 145) | 130 (114, 140) | 128 (114, 142) | 130 (120, 144) | 131 (120, 145) |
| Diastolic blood pressure | 78 (70, 85) | 70 (63, 87) | 75 (67, 82) | 78 (70, 85) | 80 (70, 88) |
| Saturation (%) | 95.0 (91.0, 97.0) | 97.0 (92.0, 98.0) | 95.0 (91.5, 97.0) | 95.0 (92.0, 97.0) | 94.0 (91.0, 97.0) |

Abbreviations: BMI: Body mass index; COPD: Chronic obstructive pulmonary disease; IQR: Inter quartile range

**Supplementary Table 2.** Characteristics of study participants by BMI categories, women.

| Group | Women | BMI <18.5 | BMI 18.5 to <25 | BMI 25 to <30 | BMI >30 |
| --- | --- | --- | --- | --- | --- |
| N | 2,986 | 130 | 919 | 916 | 1,021 |
| Age, years (IQR) | 70 (55, 82) | 81 (70, 88) | 76 (58, 86) | 71 (55, 82) | 63 (51, 75) |
| **Age group, n (%)** |  |  |  |  |  |
| 18 to 54 | 741 (25) | 14 (11) | 198 (22) | 216 (24) | 313 (31) |
| 55 to 64 | 487 (16) | 10 (7.7) | 94 (10) | 151 (16) | 232 (23) |
| 65 to 74 | 520 (17) | 20 (15) | 143 (16) | 154 (17) | 203 (20) |
| 75 to 84 | 650 (22) | 36 (28) | 223 (24) | 209 (23) | 182 (18) |
| 85 to 110 | 588 (20) | 50 (38) | 261 (28) | 186 (20) | 91 (8.9) |
| **Comorbidities, n (%)** |  |  |  |  |  |
| Hypertension | 1,355 (45) | 60 (46) | 423 (46) | 407 (44) | 465 (46) |
| Dyslipidemia | 645 (22) | 24 (18) | 186 (20) | 206 (23) | 229 (22) |
| Diabetes type I | 70 (2.3) | 1 (0.8) | 20 (2.2) | 21 (2.3) | 28 (2.7) |
| Diabetes type II | 573 (19) | 9 (6.9) | 136 (15) | 160 (17) | 268 (26) |
| Obesity | 457 (15) | 1 (0.8) | 17 (1.8) | 85 (9.3) | 354 (35) |
| Cardiovascular disease | 294 (9.9) | 17 (13) | 102 (11) | 97 (11) | 78 (7.6) |
| Atrial fibrillation | 378 (13) | 22 (17) | 141 (15) | 108 (12) | 107 (10) |
| Heart Failure | 301 (10) | 17 (13) | 103 (11) | 78 (8.5) | 103 (10) |
| COPD | 247 (8.3) | 30 (23) | 98 (11) | 57 (6.2) | 62 (6.1) |
| Asthma | 381 (13) | 8 (6.2) | 100 (11) | 123 (13) | 150 (15) |
| Chronic kidney disease | 187 (6.3) | 11 (8.5) | 73 (7.9) | 47 (5.1) | 56 (5.5) |
| Ischemic stroke | 108 (3.6) | 5 (3.8) | 41 (4.5) | 28 (3.1) | 34 (3.3) |
| Dementia | 105 (3.5) | 12 (9.2) | 44 (4.8) | 28 (3.1) | 21 (2.1) |
| **Initial vital signs, median (IQR)** |  |  |  |  |  |
| Heart rate | 83 (74, 90) | 81 (74, 90) | 81 (72, 89) | 82 (74, 90) | 85 (76, 91) |
| Temperature | 37.40 (36.80, 38.20) | 37.10 (36.60, 37.77) | 37.30 (36.70, 38.10) | 37.50 (36.80, 38.20) | 37.60 (37.00, 38.40) |
| Respiratory rate | 22.0 (18.0, 26.0) | 18.5 (16.0, 24.0) | 20.0 (17.5, 24.0) | 22.0 (18.0, 26.0) | 22.0 (20.0, 27.0) |
| Systolic blood pressure | 130 (117, 146) | 132 (115, 148) | 126 (114, 148) | 130 (116, 147) | 130 (119, 145) |
| Diastolic blood pressure | 76 (67, 84) | 77 (66, 85) | 75 (65, 82) | 76 (67, 85) | 77 (68, 84) |
| Saturation (%) | 95.0 (92.0, 97.0) | 95.0 (91.8, 98.0) | 96.0 (93.0, 98.0) | 95.0 (92.0, 97.0) | 94.0 (91.0, 97.0) |

Abbreviations: BMI: Body mass index; COPD: Chronic obstructive pulmonary disease; IQR: Inter quartile range

**Supplementary Table 3.** Analysis of interactions between sex and clinical variables for intensive care as outcome.

|  | **Multivariable** | | | **Multivariable, Interaction with Sex** | | | |
| --- | --- | --- | --- | --- | --- | --- | --- |
|  | **OR** | **95% CI** | **p-value** | **OR** | **95% CI** | **p-value** | **p-value interaction** |
| **BMI levels** |  |  |  |  |  |  |  |
| <18.5 | 1.00 | 0.59, 1.70 | >0.9 | 1.95 | 1.05, 3.64 | **0.035** | **0.048** |
| 18.5 to <25 | 1.00 | — |  | 1.00 | — | — | — |
| 25 to 30 | 1.11 | 0.92, 1.35 | 0.3 | 1.31 | 0.92, 1.85 | 0.13 | 0.13 |
| >30 | 1.42 | 1.17, 1.72 | **<0.001** | 2.00 | 1.45, 2.75 | **<0.001** | **0.011** |
| **Age group** |  |  |  |  |  |  |  |
| 18 to 54 | 0.73 | 0.59, 0.89 | **0.002** | 0.74 | 0.60, 0.90 | **0.003** |  |
| 55 to 64 | 1.00 | — |  | 1.00 | — |  |  |
| 65 to 74 | 1.12 | 0.91, 1.37 | 0.3 | 1.12 | 0.91, 1.37 | 0.3 |  |
| 75 to 84 | 0.73 | 0.58, 0.92 | **0.008** | 0.72 | 0.58, 0.91 | **0.005** |  |
| >85 |  |  |  | 0.36 | 0.26, 0.50 | **<0.001** |  |
| **Male sex** | 1.49 | 1.28, 1.74 | **<0.001** | 2.04 | 1.49, 2.79 | **<0.001** |  |
| **Diabetes type 1** | 0.98 | 0.64, 1.50 | >0.9 | 0.94 | 0.62, 1.44 | 0.8 |  |
| **Diabetes type 2** | 0.96 | 0.78, 1.18 | 0.7 | 0.94 | 0.77, 1.15 | 0.6 |  |
| **Dyslipidemia** | 0.90 | 0.73, 1.11 | 0.3 | 0.92 | 0.76, 1.13 | 0.4 |  |
| **Hypertension** | 1.20 | 1.00, 1.44 | 0.056 | 1.20 | 1.01, 1.43 | **0.044** |  |
| **Cardiovascular disease** | 1.10 | 0.85, 1.43 | 0.5 | 1.05 | 0.83, 1.34 | 0.7 |  |
| **Atrial fibrillation** | 0.63 | 0.47, 0.85 | **0.002** | 0.70 | 0.54, 0.91 | **0.008** |  |
| **Heart Failure** | 0.93 | 0.67, 1.30 | 0.7 | 0.98 | 0.73, 1.32 | >0.9 |  |
| **COPD** | 0.93 | 0.69, 1.26 | 0.6 | 0.92 | 0.69, 1.23 | 0.6 |  |
| **Asthma** | 1.16 | 0.91, 1.48 | 0.2 | 1.15 | 0.91, 1.46 | 0.2 |  |
| **Chronic kidney disease** | 1.17 | 0.85, 1.61 | 0.3 | 1.04 | 0.77, 1.40 | 0.8 |  |
| **Stroke** | 1.03 | 0.68, 1.57 | 0.9 | 0.91 | 0.62, 1.35 | 0.7 |  |
| **Dementia** | 0.29 | 0.12, 0.72 | **0.008** | 0.36 | 0.18, 0.70 | **0.003** |  |

Abbreviations: BMI: Body mass index; COPD: Chronic obstructive pulmonary disease; IC: intensive care; OR: odds ratio; CI: confidence interval

**Supplementary Table 4.** Analysis of interactions between sex and clinical variables for death as outcome.

|  | **Multivariable** | | | **Multivariable, Interaction with Sex** | | | |
| --- | --- | --- | --- | --- | --- | --- | --- |
|  | **OR** | **95% CI** | **p-value** | **OR** | **95% CI** | **p-value** | **p-value interaction** |
| **BMI levels** |  |  |  |  |  |  |  |
| <18.5 | 1.22 | 0.85, 1.75 | 0.3 | 1.72 | 0.89, 3.32 | 0.10 | 0.2 |
| 18.5 to <25 | 1.00 | — |  | 1.00 | — | — | — |
| 25 to 30 | 0.92 | 0.77, 1.08 | 0.3 | 0.75 | 0.50, 1.13 | 0.2 | 0.5 |
| >30 | 0.91 | 0.74, 1.10 | 0.3 | 0.96 | 0.66, 1.40 | 0.8 | 0.5 |
| **Age group** |  |  |  |  |  |  |  |
| 18 to 54 | 0.29 | 0.18, 0.48 | **<0.001** | 0.30 | 0.18, 0.49 | **<0.001** |  |
| 55 to 64 | 1.00 | — |  | 1.00 | — |  |  |
| 65 to 74 | 2.77 | 2.04, 3.76 | **<0.001** | 2.71 | 1.99, 3.69 | **<0.001** |  |
| 75 to 84 | 5.06 | 3.77, 6.80 | **<0.001** | 4.74 | 3.51, 6.40 | **<0.001** |  |
| >85 | 9.71 | 7.13, 13.2 | **<0.001** |  |  |  |  |
| **Male sex** | 1.72 | 1.47, 2.01 | **<0.001** | 1.54 | 1.11, 2.14 | **0.010** |  |
| **Diabetes type 1** | 0.65 | 0.42, 0.98 | **0.041** | 0.64 | 0.39, 1.04 | 0.074 |  |
| **Diabetes type 2** | 1.33 | 1.12, 1.59 | **0.001** | 1.25 | 1.01, 1.54 | **0.040** |  |
| **Dyslipidemia** | 0.90 | 0.76, 1.07 | 0.2 | 0.95 | 0.77, 1.18 | 0.7 |  |
| **Hypertension** | 1.21 | 1.02, 1.44 | **0.030** | 1.26 | 1.02, 1.57 | **0.033** |  |
| **Cardiovascular disease** | 1.12 | 0.93, 1.36 | 0.2 | 1.05 | 0.82, 1.34 | 0.7 |  |
| **Atrial fibrillation** | 0.94 | 0.78, 1.14 | 0.5 | 0.97 | 0.76, 1.25 | 0.8 |  |
| **Heart Failure** | 1.64 | 1.33, 2.02 | **<0.001** | 1.77 | 1.34, 2.33 | **<0.001** |  |
| **COPD** | 1.16 | 0.92, 1.45 | 0.2 | 1.23 | 0.94, 1.61 | 0.13 |  |
| **Asthma** | 0.97 | 0.75, 1.24 | 0.8 | 1.01 | 0.74, 1.36 | >0.9 |  |
| **Chronic kidney disease** | 1.48 | 1.18, 1.85 | **<0.001** | 1.62 | 1.21, 2.16 | **0.001** |  |
| **Stroke** | 1.20 | 0.91, 1.58 | 0.2 | 1.17 | 0.81, 1.69 | 0.4 |  |
| **Dementia** | 2.34 | 1.78, 3.08 | **<0.001** | 3.28 | 2.22, 4.85 | **<0.001** |  |

Abbreviations: BMI: Body mass index; COPD: Chronic obstructive pulmonary disease; IC: intensive care; OR: odds ratio; CI: confidence interval

**Supplementary Table 5.** Individuals excluded because of missing BMI.

|  | Missing BMI |
| --- | --- |
| N | 2 432 |
| Age, years (IQR) | 60 (46, 76) |
| **Age group, n (%)** |  |
| 18 to 54 | 991 (41) |
| 55 to 64 | 453 (19) |
| 65 to 74 | 365 (15) |
| 75 to 84 | 298 (12) |
| 85 to 110 | 325 (13) |
| **Sex, n (%)** |  |
| Women | 1 079 (44) |
| **Comorbidities, n (%)** |  |
| Hypertension | 386 (16) |
| Dyslipidemia | 154 (6.4) |
| Diabetes type I | 25 (1.0) |
| Diabetes type II | 187 (7.7) |
| Obesity | 79 (3.3) |
| Cardiovascular disease | 93 (3.8) |
| Atrial fibrillation | 106 (4.4) |
| Heart Failure | 54 (2.2) |
| COPD | 43 (1.8) |
| Asthma | 77 (3.2) |
| Chronic kidney disease | 43 (1.8) |
| Ischemic stroke | 31 (1.3) |
| Dementia | 89 (3.7) |
| **Initial vital signs, median (IQR)** |  |
| Heart rate | 84 (75, 91) |
| Temperature | 37.70 (36.90, 38.40) |
| Respiratory rate | 22 (19, 26) |
| Systolic blood pressure | 130 (117, 143) |
| Diastolic blood pressure | 78 (70, 85) |
| Oxygen saturation (%) | 95.0 (92.0, 97.0) |
| **Deaths** | 305 (12.5) |
| **Intensive care** | 222 (9.1) |

Abbreviations: BMI: body mass index; COPD: chronic obstructive pulmonary disease; IQR: inter quartile range

**Supplementary Table 6.** Sensitivity analysis, multivariable adjusted odds ratios of death from COVID-19 among regular care treated patients.

|  | **Adjusted by Age, men only** | | | **Adjusted by age, women only** | | |
| --- | --- | --- | --- | --- | --- | --- |
| **BMI levels** | **OR** | **95% CI** | **p-value** | **OR** | **95% CI** | **p-value** |
| <18.5 | 1.14 | 0.63, 2.06 | 0.7 | 1.16 | 0.69, 1.97 | 0.6 |
| 18.5 to <25 | 1.00 | — |  | 1.00 | — |  |
| 25 to 30 | 1.02 | 0.81, 1.29 | 0.9 | 0.79 | 0.58, 1.08 | 0.14 |
| >30 | 0.96 | 0.73, 1.27 | 0.8 | 0.80 | 0.57, 1.13 | 0.2 |
| **Age group** |  |  |  |  |  |  |
| 18 to 54 | 0.29 | 0.14, 0.59 | **<0.001** | 0.44 | 0.18, 1.08 | 0.073 |
| 55 to 64 | 1.00 | — |  | 1.00 | — |  |
| 65 to 74 | 3.27 | 2.11, 5.06 | **<0.001** | 3.49 | 1.80, 6.76 | **<0.001** |
| 75 to 84 | 6.16 | 4.03, 9.40 | **<0.001** | 5.01 | 2.65, 9.45 | **<0.001** |
| >85 | 13.7 | 8.77, 21.3 | **<0.001** | 8.33 | 4.39, 15.8 | **<0.001** |
| **Diabetes type 1** | 0.87 | 0.52, 1.46 | 0.06 | 0.33 | 0.11, 0.97 | **0.045** |
| **Diabetes type 2** | 1.36 | 1.07, 1.73 | **0.011** | 1.46 | 1.07, 2.00 | **0.017** |
| **Dyslipidemia** | 0.87 | 0.68, 1.10 | 0.2 | 0.85 | 0.62, 1.16 | 0.3 |
| **Hypertension** | 1.18 | 0.93, 1.49 | 0.2 | 1.19 | 0.87, 1.63 | 0.3 |
| **Cardiovascular disease** | 1.15 | 0.89, 1.48 | 0.3 | 1.01 | 0.70, 1.46 | >0.9 |
| **Atrial fibrillation** | 0.89 | 0.69, 1.15 | 0.4 | 0.97 | 0.69, 1.36 | 0.9 |
| **Heart Failure** | 1.57 | 1.18, 2.08 | **0.002** | 1.94 | 1.35, 2.79 | **<0.001** |
| **COPD** | 1.05 | 0.76, 1.46 | 0.7 | 1.08 | 0.72, 1.61 | 0.7 |
| **Asthma** | 0.70 | 0.46, 1.06 | 0.091 | 0.87 | 0.59, 1.30 | 0.5 |
| **Chronic kidney disease** | 1.38 | 1.00, 1.82 | **0.047** | 1.82 | 1.22, 2.70 | **0.003** |
| **Stroke** | 1.50 | 1.05, 2.15 | **0.026** | 0.81 | 0.47, 1.40 | 0.5 |
| **Dementia** | 3.44 | 2.38, 4.97 | **<0.001** | 1.50 | 0.93, 2.43 | 0.10 |

Abbreviations: BMI: Body mass index; CI: confidence interval; COPD: Chronic obstructive pulmonary disease; OR: odds ratio.

**Supplementary Figure 2.** Age adjusted odds ratios (95% CI) of death by unit of increase in BMI with BMI 20 kg/m^2^ as a reference, for men (left) and women (right) among RC patients only.

Abbreviations: BMI: Body mass index; RC: regular care.

**
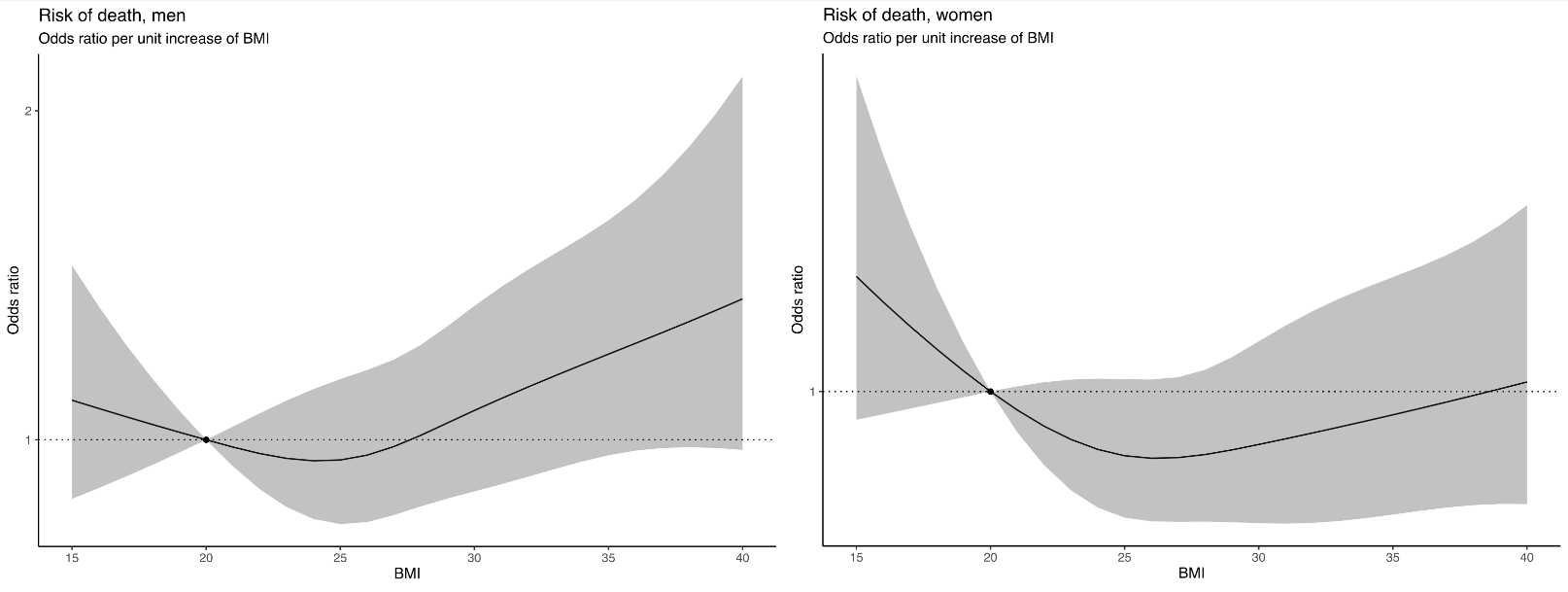
**
